# The Gut Microbiota and Autism Spectrum Disorders

**DOI:** 10.3389/fncel.2017.00120

**Published:** 2017-04-28

**Authors:** Qinrui Li, Ying Han, Angel Belle C. Dy, Randi J. Hagerman

**Affiliations:** ^1^Department of Pediatrics, Peking University First HospitalBeijing, China; ^2^School of Medicine and Public Health, Ateneo de Manila UniversityQuezon City, Philippines; ^3^MIND Institute, University of California Davis Medical CenterSacramento, CA, USA; ^4^Department of Pediatrics, University of California Davis Medical CenterSacramento, CA, USA

**Keywords:** autism spectrum disorder (ASD), gut microbiota, brain-gut axis, probiotics, fecal microbiota transplantation (FMT)

## Abstract

Gastrointestinal (GI) symptoms are a common comorbidity in patients with autism spectrum disorder (ASD), but the underlying mechanisms are unknown. Many studies have shown alterations in the composition of the fecal flora and metabolic products of the gut microbiome in patients with ASD. The gut microbiota influences brain development and behaviors through the neuroendocrine, neuroimmune and autonomic nervous systems. In addition, an abnormal gut microbiota is associated with several diseases, such as inflammatory bowel disease (IBD), ASD and mood disorders. Here, we review the bidirectional interactions between the central nervous system and the gastrointestinal tract (brain-gut axis) and the role of the gut microbiota in the central nervous system (CNS) and ASD. Microbiome-mediated therapies might be a safe and effective treatment for ASD.

Autism spectrum disorder (ASD) constitutes a group of brain developmental disorders, and it is defined by stereotyped behavior and deficits in communication and social interaction. ASD has a significant influence on the development of children and on society. In 2012, the estimated prevalence of ASD was 14.6 per 1,000 children aged 8 years, and the prevalence was significantly higher in boys (23.6 per 1,000) than that in girls (5.3 per 1,000) (Christensen et al., 2016). The cost of caring for a child with ASD but without an intellectual disability is £0.92 million in the United Kingdom and $1.4 million in the United States. The main costs associated with the care of children with ASD are special education services and a loss of parental productivity (Buescher et al., 2014). Therefore, the economic effects of ASD have prompted researchers to search for effective interventions. However, identifying the exact etiology and pathology of ASD is difficult, and available effective therapies are limited (Rossignol and Frye, 2012). Previous studies have focused on genetic causes, dysregulation of the immune system, inflammation, exposure to environmental toxicants, and the gut microbiota (Fakhoury, 2015). The heritability of ASD and autistic disorder was approximately 50% among Swedish children, suggesting that both genetic and environmental factors play important roles in the development of ASD (Hallmayer et al., 2011; Sandin et al., 2014). Accumulating evidence demonstrates that gastrointestinal (GI) symptoms, such as abdominal pain, gaseousness, diarrhea, constipation and flatulence, are a common comorbidity in patients with ASD (Chaidez et al., 2014). A study by Gorrindo et al. identified constipation as the most common symptom (85%) in children with ASD according to parental reports and evaluations by pediatric gastroenterologists (Gorrindo et al., 2012). The prevalence of GI symptoms ranges from 23 to 70% in children with ASD (Chaidez et al., 2014). Furthermore, the observed GI symptoms are associated with the severity of ASD (Adams et al., 2011; Gorrindo et al., 2012). Although these studies did not show a cause-effect relationship between GI symptoms and ASD, the findings suggest that the gut plays an important role in the etiology of ASD. The gut consists of millions of microbiota, and we hypothesize that the microbiota and its metabolites might be involved in the pathophysiology of ASD. Several articles have reviewed the influence of the gut microbiota on the animal central nervous system (CNS) and suggested the existence of a microbiota gut-brain axis (Bienenstock et al., 2015; Mayer et al., 2015). The microbiota-gut-brain axis is likely the method of communication between the brain and the gut microbiota. This article reviews the role of the gut microbiota in the pathology of ASD. Strategies that modulate the gut microbiota might constitute a potential therapy for patients with ASD.

## Gut microbiota

The human gut consists of approximately 1 kg of bacteria, and the number of bacterial genes are in the gut is approximately about 9.9 million. The ratio of host DNA vs. microbiome DNA is 1:10. (Li et al., 2014). Our knowledge of the microbiome has excessively expanded over the last few years. For example, researchers previously believed that the *in utero* environment was sterile (Jimenéz et al., 2008; Martin et al., 2016). However, recent work has shown that the infant gut is colonized by the microbiome of the maternal vagina, anus and skin during delivery and by the environmental bacteria to which the neonate is exposed during the postpartum period (Dominguez-Bello et al., 2010; Bokulich et al., 2016; Yassour et al., 2016). As demonstrated in recent studies, the placenta and the amniotic fluid are not sterile (DiGiulio et al., 2008; Aagaard et al., 2014). In addition, the microbiome in the first meconium of mice is not sterile, indicating that the microbiome colonizes the infant gut prior to parturition (Jimenéz et al., 2008). Maternal factors, such as maternal diet and delivery mode, and postnatal factors, including antibiotics, breast-feeding, diet and host genetics, structure the neonatal microbiome in humans and animal models (Tamburini et al., 2016). Many studies have shown that a maternal high-fat diet during pregnancy decreases the level of *Bacteroides* in human neonates and diminishes the abundance of non-pathogenic *Campylobacter* in primates (Ma et al., 2014; Chu et al., 2016). Furthermore, maternal obesity during pregnancy and gestational diabetes alter the gut microbiota and might be associated with ASD in humans (Connolly et al., 2016). As shown by Buffington et al., a maternal high-fat diet induces dysbiosis and autism-like phenotypes in mice, and *Lactobacillus reuteri* restores these alternations (Buffington et al., 2016). The birth mode and antibiotics also shape the gut microbiota (Bokulich et al., 2016). The gut microbiota of infants who were delivered vaginally resembles their mother's vaginal microbiota, which is dominated by *Lactobacillus, Prevotella*, or *Sneathia* spp., and the gut microbiota of babies who were born by Cesarean section is similar to their mother's skin microbiota, which is dominated by *Staphylococcus, Corynebacterium*, and *Propionibacterium* spp. (Dominguez-Bello et al., 2010). As shown by Yassour et al., the composition of the microbiota of children who were treated with antibiotics during the first 3 years of life is less diverse in terms of both bacterial species and strains (Yassour et al., 2016). A population-based cohort study revealed the use of various antibiotics during pregnancy as a potential risk factor for ASD/infantile autism (Atladóttir et al., 2012). The early feeding pattern also influences the gut microbiota of infants and is associated with ASD. Formula-fed infants present an increased species richness accompanied by an overrepresentation of *Clostridium difficile* compared with breast-fed infants (Azad et al., 2013). Breast-feeding for more than 6 months is associated with a lower risk of developing ASD (Schultz et al., 2006). Penn et al. studied infants with an older sibling diagnosed with ASD in the San Diego area and found that breast-feeding might protect the infants against GI symptoms (Penn et al., 2016). As an individual's diet diversifies with increasing age, the gut microbiota gradually stabilizes (Koenig et al., 2011; Yatsunenko et al., 2012; Tamburini et al., 2016). A diet with lean ground beef increases the diversity of the gut microbiota and improves both working and reference memory in mice compared with a standard rodent chow diet, suggesting a correlation between diet-induced shifts in the gut microbiota and animal behaviors (Li et al., 2009a). Environmental exposure also affects the microbiota of humans. Furthermore, dysbiosis of the gut microbiota is associated with several disorders in children, such as abnormal behaviors, Crohn's disease, obesity and inflammatory bowel disease (IBD) (Ajslev et al., 2011; Cryan and Dinan, 2012; Jostins et al., 2012). In summary, the gut microbiota plays important roles in human physiology and pathology.

## Relationship between ASD and gut microbiota

Gastrointestinal (GI) symptoms are prominent in ASD individuals. Wang et al., found more GI syndromes, including constipation (20%) and diarrhea (19%), in children with ASD than in their unaffected siblings (42 vs. 23%, respectively) (Wang et al., 2011). Two meta-analyses showed similar results in children with ASD (Coury et al., 2012; McElhanon et al., 2014). Patients with ASD who present GI symptoms might display significant behavioral manifestations, such as anxiety, self-injury and aggression (Buie et al., 2010). Accumulating evidence demonstrates that the gut microbiota is directly or indirectly associated with ASD symptoms, in part by influencing the immune system and metabolism (De Angelis et al., 2015; Mead and Ashwood, 2015). A higher percentage of abnormal intestinal permeability was observed in 36.7% of patients with ASD and their relatives (21.2%) compared with control children (4.8%) (de Magistris et al., 2010). An increased intestinal permeability results in a higher antigenic load from the gastrointestinal tract. Lymphocytes and ASD-associated cytokines, such as interleukin-1β (IL-1β), IL-6, interferon-γ (IFN-γ), and tumor necrosis factor-a (TNF-α), are present in the circulation and cross the blood-brain barrier (BBB). Subsequently, IL-1β and TNF-α bind to brain endothelial cells and induce immune responses in the brain (Li et al., 2009b; Ashwood et al., 2011; de Theije et al., 2011). Alterations in the composition of the gut microbiota and their metabolic products are commonly observed in patients with ASD and in animal models of ASD (de Magistris et al., 2010; Borre et al., 2014; Kushak et al., 2016). Hsiao et al. observed gastrointestinal barrier defects and microbiota alterations in a mouse model displaying features of ASD. They found that bacteria belonging to *Porphyromonadaceae, Prevotellaceae*, unclassified *Bacteroidales*, and *Lachnospiraceae* were more abundant in offspring of mothers with maternal immune activation (MIA) than in control offspring, whereas *Ruminococcaceae, Erysipelotrichaceae*, and *Alcaligenaceae* were more abundant in the latter (Hsiao et al., 2013). As shown in mice, the anti-epileptic drug valproic acid (VPA), when used by the mother during pregnancy, induces autistic-like social behaviors in the offspring accompanied by alterations in *Bacteroidetes* and *Firmicutes* (de Theije et al., 2014b). Compared with the gut microbiota of children without ASD, the gut microbiota of children with ASD is less diverse and exhibits lower levels of *Bifidobacterium* and *Firmicutes* and higher levels of *Lactobacillus, Clostridium, Bacteroidetes, Desulfovibrio, Caloramator* and *Sarcina* (Finegold et al., 2002, 2010; Adams et al., 2011; Finegold, 2011; De Angelis et al., 2013). Children with autism who present GI symptoms have lower abundances of the genera *Prevotella, Coprococcus*, and unclassified *Veillonellaceae* than that found in GI symptom-free neurotypical children (Kang et al., 2013). Fecal samples from children with ASD also have higher levels of the *Clostridium histolyticum* group (*Clostridium* clusters II and I) compared with samples from unrelated healthy children (Parracho et al., 2005). The non-autistic sibling group presents an intermediate level of *Clostridium histolyticum* that does not significantly differ from the ASD group. *Clostridium* can produce neurotoxins and might exert systemic effects (Parracho et al., 2005). The reduction of *Clostridium* yields significant improvements in children with ASD (Sandler et al., 2000). Additionally, children with ASD present alterations in their levels of *Bifidobacterium, Prevotella*, and *Sutterella* (Wang et al., 2013). *Ruminococcus torques* has been associated with functional GI disorder (Joossens et al., 2011). Infants who were delivered by Cesarean section (CS) are at higher risk of developing ASD (odds ratio of 1.23) than infants delivered vaginally (Curran et al., 2015). Children with ASD have a history of using significantly more antibiotics (Niehus and Lord, 2006; Shultz et al., 2008; Atladóttir et al., 2010, 2012). Thus, early life events that can alter the composition of the microbial community, such as delivery mode and antibiotic exposure, are risk factors for ASD. However, some studies have not found an association between ASD and the gut microbiota. A study of 59 ASD individuals and 44 normal siblings via targeted qPCR found no significant difference in *Sutterella, Prevotella* or total *Bacteroidetes* composition between them (Son et al., 2015).

Although many studies have demonstrated alternations to the bacterial gut microbiota in ASD patients, fewer studies have evaluated the relationships between gut fungi and ASD. The yeast in gut (particularly *Candida*. *albicans*) result in absorbing carbohydrates and minerals fewer and releasing higher toxins. Kantarcioglu et al. isolated 338 yeast strains from 415 stool samples of ASD individuals. Among the yeast strain, 81.4% were *Candida* (particularly *Candida*. *albicans*). Lower yeast isolated rate (19.6%) were identified in non-autistic healthy volunteers. *Candida*. *krusei* and *Candida*. *glabrata* were not found in healthy children (De Angelis et al., 2013; Kantarcioglu et al., 2016). Strati et al., found a significant increase in the *Firmicutes*/*Bacteroidetes* ratio in autistic subjects relative to normal subjects. They also found that *Candida* was two times more abundant in autistic individuals than in normal individuals (Strati et al., 2017). The proliferation of *Candida* can be inhibited by IL-17, IL-22, which modulated by some species of *Lactobacillus* through tryptophan-derived aryl hydrocarbon receptor ligands (Zelante et al., 2013). *Candida* can releases ammonia and toxins that can induce autistic behaviors (Burrus, 2012; Iovene et al., 2017). The alterations of the bacterial microbiota in ASD individuals result in the expansion of *Candida*, which would worsen the dysbiosis and induce the abnormal behaviors. In summary, the role of gut fungal in ASD still need more large samples studies.

## Potential relationships between the microbiota and ASD (the gut-brain axis)

**Figure 1:**
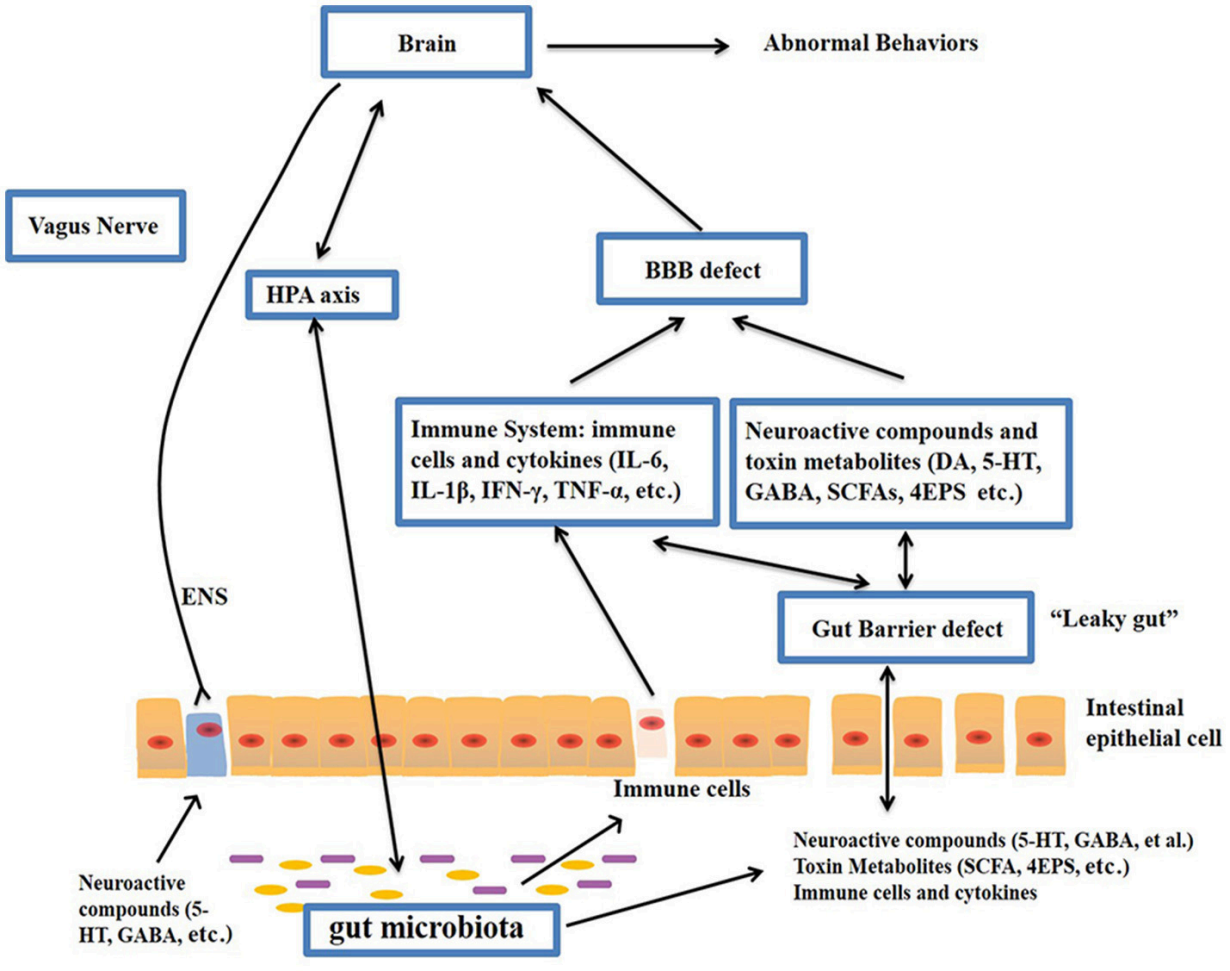
**Potential relationships between the microbiota and ASD (the gut-brain axis)**. The production of metabolites, such as SCFAs and toxin metabolites, by certain microbiota (e.g., *Lactobacillus*) can cross the “leaky gut” to affect brain function. Some microbiota can produce neuroactive compounds (e.g., 5-HT and GABA) that cross the “leaky gut” and influence brain function and induce abnormal behaviors. These neuroactive compounds can directly influence the HPA axis and increase circulating levels of cortisol. Metabolites, certain microbiota and neuroactive compounds can activate enteric neurons and affect brain function through the vagus nerve. Some microbiota and metabolites can activate gut immune cells, which can release cytokines into circulation. 4-EPS, 4-ethylphenyl sulfate; 5-HT, serotonin; HPA, hypothalamic–pituitary–adrenal; SCFAs, short-chain fatty acids; BBB, blood-brain barrier; 5-HT, 5-hydroxytryptamine; ENS, enteric nervous system; GABA, γ-aminobutyric acid; DA, dopamine.

The gut–brain axis is regarded as a pathway of communication between the gut and the brain, and it is a bidirectional communication system. An increasing body of evidence shows that the gut-brain axis participates in the pathogenesis of ASD. The gut microbiota influences brain function through the neuroendocrine, neuroimmune and autonomic nervous systems and via microbiotic toxin production (Grenham et al., 2011; Mayer, 2011; Figure 1). The mucosa of the gastrointestinal tract contains millions of neurons, which constitute the enteric nervous system (ENS) and regulate gastrointestinal functions. Therefore, the gut is considered as a “second brain.” Figure 1 outlines the potential relationships between the gut microbiota and ASD.

A fundamental factor underlying the relationships between ASD and the gut is the increased permeability of the intestinal tract of ASD individuals, referred to as a “leaky gut” (Quigley, 2016). Previous studies have demonstrated that ASD animals present defects in the GI barrier, resulting in the entry of the toxins and bacterial products into the bloodstream, which influence brain function (Onore et al., 2012; Hsiao et al., 2013). For example, lipopolysaccharide (LPS), components of the cell wall of gram-negative bacteria, is increased in the serum of ASD compared with healthy individuals and is associated with impaired social behavioral scores (Emanuele et al., 2010). Fiorentino et al., found that the integrity of both the gut barrier and the BBB were impaired in ASD individuals, as evidenced by increased levels of claudin (CLDN)-5, CLDN-12, CLDN-3, and MMP-9 in the ASD brain and decreased levels of intestinal tight junction components (CLDN-1, OCLN, TRIC) in ASD individuals compared with controls. (Fiorentino et al., 2016). Intestinal permeability, measured by the lactulose: mannitol test, has been shown to be increased in autistic children compared with healthy controls (de Magistris et al., 2010). Furthermore, germ-free (GF) mice display increased BBB permeability. Bacterial products (e.g., acetate and propionate) can enhance the integrity of the BBB (Braniste et al., 2014).

### Gut microbiota-mediated metabolites

Gut microbiota-mediated metabolites, such as short-chain fatty acids (SCFAs), phenol compounds, and free amino acids (FAA), affect ASD-like behaviors through the vagal pathways (Shimmura et al., 2011; Macfabe, 2012; Persico and Napolioni, 2013; Forsythe et al., 2014).

SCFAs, including acetic acid (AA), proprionic acid (PPA), butyrate, isobutyric acid, valeric acid and isovaleric acid, are principal products of the gut bacterial fermentation of non-digestible carbohydrates (Al-Lahham et al., 2010) and provide benefits to the host, including improvements in glucose and energy homeostasis and reductions in body weight and the risk of colon cancer (Mariadason et al., 2000; De Vadder et al., 2014; Chambers et al., 2015). According to several studies, SCFAs play a critical role in patients with ASD. A study by Wang et al., detected higher concentrations of total SCFAs and ammonia in fecal matter from children with autism compared with controls (Wang et al., 2012). PPA, a short-chain fatty acid that is mainly produced by *Clostridia, Bacteroidetes*, and *Desulfovibrio*, can cross the BBB and induce ASD-like behaviors (MacFabe et al., 2007, 2011; Shultz et al., 2008; Ossenkopp et al., 2012). As shown by Thomas et al., the intracerebroventricular administration of high doses of PPA provokes some autistic-like behaviors in mice (Thomas et al., 2012), and the intraventricular administration of PPA to rats results in hyperactivity, repetitive behaviors and abnormal motor movements, similar to the behavioral and electrographic changes detected in humans with ASD (MacFabe et al., 2007). PPA leads to impaired social behavior in rats, likely by altering some neurotransmitters, such as dopamine and serotonin (Mitsui et al., 2005). Butyrate can modulate synthesis of the neurotransmitters dopamine, norepinephrine and epinephrine by altering expression of the tyrosine hydroxylase gene (DeCastro et al., 2005). Butyrate also inhibits histone deacetylases and possesses anti-inflammatory effects (Cleophas et al., 2016). Researchers have found lower serum levels of 4-ethylphenylsulfate (4EPS), indolepyruvate, glycolate, imidazole propionate and N-acetylserine in a MIA model of ASD. Naïve wild-type mice treated with 4EPS potassium salt presents ASD-related behavior (Hsiao et al., 2013). FAA, derived from the hydrolysis of proteins and peptides, has also been found to be associated with ASD. De Angelis et al., found that the level of total FAA in fecal samples was higher in children with autism than that in heathy children. In addition, the levels of Asp, Ser, Glu, Gly, Ala, Val, Ile, Phe, His, Tpr, Lys, and Pro in fecal samples were higher in the children with autism (De Angelis et al., 2015). Glu, a neurotransmitters in the CNS, has been found to be associated with some neuropsychiatric disorders and ASD (Sheldon and Robinson, 2007; Shimmura et al., 2011). Noto et al., found that tryptophan, a neurotransmitter precursor, was increased in the urine of ASD patients. In addition, they found increased levels of tryptophan fragments in urine and increased tryptophan degradation, which are also found in depression and mental retardation (Noto et al., 2014). When compared with urine samples from control children, the urine samples from children with ASD contain higher levels of 2-(4-hydroxyphenyl) propionate and taurocholenate sulfate and lower levels of 3-(3-hydroxyphenyl) propionate and 5-amino-valerate (Ming et al., 2012). 3-(3-hydroxyphenyl)-3-hydroxypropanoic acid, a phenylalanine metabolite of *Clostridia* spp. was shown to be increased in the urine of ASD patients and is associated with autistic behaviors in animals (Shaw, 2010).

### The immune system pathways

The gut can also communicate with the brain through immunological pathways. Many studies have shown increased levels of pro-inflammatory cytokines, such as IL-1β, IL-6, IL-8, and IL-12p40, in the plasma of ASD individuals (Ashwood et al., 2011). Immune responses to toxins produced by pathogenic microbiota and focal inflammation increase gut permeability. An impaired intestinal barrier is observed in response to infection or stress, which allows the translocation of the gut bacteria across the intestinal wall and into the mesenteric lymphoid tissue, where they activate the immune system through mucosal immune cells (Dicksved et al., 2012). The activated immune system releases inflammatory cytokines and activates the vagal system, which in turn regulates CNS activity (Yarandi et al., 2016). Konsman et al., found peripheral cytokines to induce behavioral depression through the vagus nerve (Konsman et al., 2000). Moreover, metabolic compounds, such as lipopolysaccharide (LPS) produced by gut microbiota, are absorbed into the blood through an impaired gut wall and activate Toll-like receptors in the ENS and CNS (Abreu, 2010). An IgE-mediated allergic immune response in the intestine increases the 5-hydroxytryptamine (5-HT) levels and decreases the 5-hydroxyindoleacetic acid (5-HIAA) levels in the intestine. It also reduces social communication and increases repetitive behavior. It mice, these effects are accompanied by a downregulation of dopaminergic activity in the prefrontal cortex and activation of the neuroendocrine system in mice (de Theije et al., 2014b).

### Neuroactive compound pathways

The pathway via which the microbiota communicate with the brain can also involve neurotransmitters. The gut microbiota produces neuroactive compounds such as dopamine (DA), 5-HT, γ-aminobutyric acid (GABA) and histamine, which activate or inhibit central neurons through the vagus nerve (Eisenstein, 2016; Spiller and Major, 2016). Compared with specific-pathogen-free (SPF) mice with a normal gut microbiota, GF mice exhibit a significant elevation of monoamine neurotransmission (of compounds such as noradrenaline, DA and 5-HT), decreased levels of nerve growth factor-inducible clone A (NGFI-A) and brain-derived neurotrophic factor (BDNF), increased corticosterone levels, and increased anxiety-like behaviors (Diaz Heijtz et al., 2011; Neufeld et al., 2011; Clarke et al., 2013). Antibiotic-induced depletion of the gut microbiota in mice impairs learning and increases depression-like behaviors. Furthermore, the levels of CNS neurotransmitters (e.g., 5-HT, 5-hydroxyindoleacetic acid, noradrenaline, DA and the metabolite homovanillic acid) as well as the mRNA levels of the glucocorticoid receptor and corticotrophin-releasing hormone receptor 1 have been shown to be altered (Hoban et al., 2016). The gut microbiota also affects mammalian brain development and subsequent adult behavior. For instance, GF mice display lower expression of postsynaptic density protein 95 (PSD-95) and synaptophysin in the striatum, increased monoamine neurotransmission and motor activity and reduced anxiety compared with SPF mice (Diamond et al., 2011; Diaz Heijtz et al., 2011). Blood serotonin (5-HT) was the first ASD biomarker identified and is present in approximately 30% of individuals with ASD (Pare et al., 1960; Schain and Freedman, 1961; Hanley et al., 1977; Mulder et al., 2004). SERT 5-HT transporter (SERT) Ala56-knock-in mice, which display hyperserotonemia as a result of increased neuronal absorption of 5-HT, show abnormal social behaviors, nonstandard communication, and repetitive behaviors (Veenstra-VanderWeele et al., 2012). Serotonin, which is synthesized in the intestines and brain, is important for the regulation of mood and cognition (Cryan et al., 2000). Marler et al., found an association between whole-blood serotonin levels and GI symptoms in ASD individuals (Marler et al., 2016). Compared to the brains of offspring from the control mice, the brains of offspring from VPA-exposed mice present altered microbiota and lower levels of serotonin and ASD-like behavior (de Theije et al., 2014a).

## Modulation of the gut microbiota is a potential therapy for children with ASD

At present, there are no effective therapies for ASD. Parents often take their children to receive intervention that is tailored to their specific needs. It is urgent to look for risk-free and effective treatments. Accumulating evidences showed modulation of the gut microbiota is a potential therapy in children with ASD. Probiotics, prebiotics, fecal microbiota transplantation (FMT) and diet have getting considerable attention (Table 1). Probiotics may prevent intestinal inflammatory diseases by regulating intestinal tight junction protein expression and barrier function. The use of heat-killed probiotics may provide therapeutic benefit while minimizing adverse effects.

**Table 1:** **Studies of the autism spectrum disorder (ASD) treatments**.

**Model**	**Behavior Tests**	**Treatment**	**Dosage and time**	**Effects**	**Limitations**	**References**
ASD animal model	Pre-pulse inhibition, open field exploration, marble burying, social interaction and adult ultrasonic vocalizations	Probiotic:*Bacteroides fragilis*	1 × 10^10^ CFU every otherday for 6 days	Improved gut barrier integrity, normalized gut microbiota, reversed ASD-related behaviors, decreased 4EPS in serum	An animal study	Hsiao et al., 2013
33 ASD children	ATEC	Delpro® (containing *Lactocillus acidophilus, Lactobacillus casei, Lactobacillus delbruecki, Bifdobacteria longum, Bifdobacteria bifdum* and 8 mg of Del-Immune V powder).	1 × 10^8^ billion CFUs, three times daily for 6 months	88% of patients reported a decrease in total ATEC score, 48% reported a decrease in diarrhea and 52% reported a decrease in constipation	There was no control or placebo, and has a selection bias	West et al., 2013
10 autistic children, their 9 non-autistic siblings and 10 control	CARS, ADI	Probiotic containing *Lactobacillus, Bifidobacteria and Streptococci*	One capsule three times a day for 4 months.	Increased of the *Bacteroidetes/Firmicutes* ratio, normalized the amount of *Bifidobacterium* and *Lactobacillus* and decreased the TNFα levels in the feces of children with autism	Not mentioned the alternation of ASD-like behavior after probiotic treatment	Tomova et al., 2015
22 autistic children	Not mentioned	Probiotic: Lactobacillus acidophilus	5 × 10^9^ CFU/g twice daily for 2 months	Decreased DA/LA ratio in urine, improved some autistic symptoms (e.g., ability of concentration and carrying out orders)	No control group. The behavior tests are not clear	Kaluzna-Czaplinska and Blaszczyk, 2012
A 12 years old boy with ASD and severe cognitive disability	ADOS-2	Probiotic: VSL#3 contains lyophilized bifidobacteria (*Bifidobacterium breve, B. longum, B. infantis)*, lactobacilli (*Lactobacillus acidophilus, L. plantarum, L. paracasei, L. bulgaricus, L. delbrueckii subsp.)* and Streptococci (*S. thermophilus, S. salivarius subsp*.)	9 × 10^10^ cfu/g lyophilized *bifidobacteria*, 8 × 10^10^ *lactobacilli* and 20 × 10^10^ *Streptococci* for 4 weeks	Reduced the severity of abdominal symptoms and improved in autistic core symptoms	A case report. Results of this study need to be confirmed in well-controlled trials with sufficient sample-scale	Grossi et al., 2016
11 autistic children	CARS	Vancomycin and probiotic (*Lactobacillus acidophilus, L bullgaricus*, and *Bifidobacterium bifidum*) and vancomycin	Vancomycin (500 mg/day) three times/day for 8 weeks, probiotics (40 × 10^9^ CFU/mL) for 4 weeks	Short-term improvement of behavioral scores during the vancomycin treatment		Sandler et al., 2000
3 autistic child and 3 non-autistic children (in an *in vitro* gut model system.)	No	Prebiotic: galactooligosaccharide (B-GOS) consisting of GOS, lactose, glucose and galactose	2 g/daily	Increases the levels of *Bifidobacterium* spp. and increase of acetate and butyrate	*In vitro* study (gut model system)	Grimaldi et al., 2017
18 children with ASD	PGI-III, CARS, ABC, SRS, VABS-II	Microbiota Transfer Therapy (MTT): 14 days vancomycin treatment, a bowel cleanse (MoviPrep) and administered a high initial dose of SHGM	Vancomycin (40 mg/kg per day) for 2 weeks, SHGM (2.5 × 10^12^ cells/day) for 7–8 weeks	Improved both GI symptoms (e.g., constipation, diarrhea, indigestion and abdominal pain) and ASD-related symptoms, and normalized the microbiota of ASD patients	The open-label trial is not placebo controlled, blinded or randomized	Kang et al., 2017
41 ASD patients	SRS-P, CBCL	Omega-3 fatty acids (EPA + DHA)	1 g/day of omega-3 fatty acids for 12 weeks	Improved ASD core symptoms, social problems and attention problems in CBCL assessment	An open-label study	Ooi et al., 2015
38 ASD patients	PDDBI, VABS-II, PLS-4, CGI-I scale	EPA+DHA	0.75 g of EPA + DHA (1.875 ml) + 1.5 g (3.5 ml) once a day	There was no evidence for efficacy of omega-3 fatty acids on core symptom domains. There was a statistically significant difference in externalizing behaviors	Sample size is small	Mankad et al., 2015
20 ASD patients	Leiter International Performance Scale, ITPA, TOMI	A gluten-free and/or casein-free (GF/CF) diet	1 year	Reduced autistic traits (e.g., aloofness, routines and rituals); improves ASD behaviors, physiological symptoms, and social behaviors	The expectations of a positive effect of diet have influenced the results of the control group	Knivsberg et al., 2002
387 ASD children	ASD behaviors, physiological symptoms, social behaviors	A gluten-free and/or casein-free (GF/CF) diet	1 year	Improves ASD behaviors, physiological symptoms, and social behaviors	A retrospective analysis of parental report	Pennesi and Klein, 2012
BTBR mice and C57B1/6 mice	social novelty test, three-chamber sociability test, social transmission of a food preference	Ketogenic diet	3–5 weeks	Improved behavioral symptoms of ASD.	Additional research on KDs or analogous metabolism-based strategies should be considered	Ruskin et al., 2013
ASD animal model	tail-flick test, marble burying test, self-grooming evaluation, three chambers social test	Ketogenic diet	3 weeks	Prevented social deficits and stereotypies	Not measure the ketosis and glucose levels	Castro et al., 2016
C57BL/6 and BTBR mice	the three-chamber sociability test, social transmission of a food preference	ketogenic diet	10–14 days	Decreased the elevated *Akkermansia muciniphila* and decreased total host bacterial abundance in cecal and fecal matter		Newell et al., 2016
34 ASD children	CARS, ATEC, CGI	levocarnitine	50 mg/kg L-carnitine per day, for 3 months	Improved clinical measurements and blood levels of carnitine; reduced total fat mass and increased total muscle mass	The sample size is small.	Geier et al., 2011

*CFU, colony-forming units; 4EPS, 4-ethylphenylsulfate; PGI-III, The Parent Global Impressions III; CARS, The Childhood Autism Rating Scale; ABC, The Aberrant Behavior Checklist; SRS, The Social Responsiveness Scale; VABS-II The Vineland Adaptive Behavior Scale II; SHGM, Standardized Human Gut Microbiota; ITPA, Illinois Test of Psycholinguistic Abilities; TOMI, Test of Motor Impairment; PDDBI, Pervasive Developmental Disorder Behavioral Inventory; VABS-II, Vineland Adaptive Behavior Scales, Second Edition; PLS-4, Preschool Language Scale-4; CGI-I scale, Clinical Global Impression-Improvement scale; SRS-P, The Social Responsiveness Scale–Parent, CBCL, The Child Behavior Checklist; EPA, eicosapentaenoic acid; DHA, docosahexaenoic acid; ATEC, Autism treatment evaluation checklist; DSM-5, Diagnostic and Statistical Manual of Mental Disorders' (5th ed) criteria; ADOS-2, Autism Diagnostic Observation Schedule-2; ADI, Autism Diagnostic Interview*.

### Probiotics and prebiotics

Probiotics, such as the lactic acid-producing bacteria belonging to *Lactococcin, Lactobacilli, Bifidobacteria* and *Saccharomycetes*, are beneficial to the host when provided in adequate quantities. Many studies have shown that probiotics can prevent and treat a variety of diseases, such as obesity, depression, colorectal cancer and Crohn's disease, in animal models and humans (Verna and Lucak, 2010; Verma and Shukla, 2014; Sharma and Shukla, 2016; Valsecchi et al., 2016). In heathy women without gastrointestinal or psychiatric symptoms, the consumption of a fermented milk product containing *Bifidobacterium animalis* subsp *lactis, Streptococcus thermophilus, Lactobacillus bulgaricus*, and *Lactococcus lactis* subsp *lactis* results in robust alterations in activity in the brain regions that control the central processing of emotions and sensations, as observed by functional magnetic resonance imaging (Tillisch et al., 2013). Patients with irritable bowel syndrome (IBS) who were treated with *B. infantis* 35624 reported that alleviation of symptoms such as abdominal pain and distention and presented a normalized ratio of the immunomodulatory cytokines IL-10/IL-12 (O'Mahony et al., 2005; Whorwell et al., 2006). In a double-blind, placebo-controlled, randomized study, *Lactobacillus helveticus* R0052 and *Bifidobacterium longum* R0175 were administered to healthy women for 30 days, and then anxiety, depression, and 24-h free cortisol levels in the urine were then assessed. The probiotics alleviated psychological distress and the 24-h urinary cortisol levels (Messaoudi et al., 2011). In another pilot clinical study, patients with chronic fatigue syndrome received 24 billion colony-forming units of *Lactobacillus casei* strain Shirota daily for 2 months, and a significant decrease in anxiety symptoms and a significant increase in *Lactobacillus* and *Bifidobacteria* were observed (Rao et al., 2009). Probiotic, such as *Lactobacillus reuteri* and *Lactobacillus rhamnosus*, have been shown to improve barrier function via altering the expression of tight junction proteins and decreased the bacterial translocation in animal models or *in vitro* (Ulluwishewa et al., 2011; Dicksved et al., 2012; Patel et al., 2012). Healthy human administered *Lactobacillus plantarum* strain WCFS1 in the duodenum enhanced the intestinal barrier by regulating human epithelial tight-junction proteins (Karczewski et al., 2010). Patel et al., found *Lactobacillus rhamnosus* GG (LGG) promoted intestinal barrier function maturation by inducing claudin 3 expression (Patel et al., 2012).

The probiotic/prebiotic can normalize the gut microbiota, enhance gut barrier and relieve the ASD-like behaviors in animal models or ASD patients. As shown by a study of Hsiao et al., treatment with *Bacteroides fragilis* reduced gut permeability, altered the composition of the gut microbiota and decreased ASD-like behaviors in a rodent model of ASD (Hsiao et al., 2013). West et al., found that the daily administration of the probiotic and immune modulator co-formulation Delpro® significantly improved GI and ASD symptoms (West et al., 2013). Supplementation with a probiotic containing *Lactobacillus, Bifidobacteria* and *Streptococci* normalizes the *Bacteroidetes/Firmicutes* ratio, and the amounts of *Desulfovibrio* spp. and *Bifidobacterium* spp. in the feces of children with ASD are similar to those found in samples from their non-autistic siblings or unrelated heathy controls. They also found the Autism Diagnostic Interview (ADI) restricted/repetitive behavior subscale score has associated with the amount of *Desulfovibrio* spp. (Tomova et al., 2015). However, the authors did not assess the alteration of ASD behavior after probiotic treatment. According to a cohort study, oral supplementation with *Lactobacillus acidophilus* twice daily for 2 months decreases the D-arabinitol levels in the urine of children with ASD and improves their ability to follow directions, as demonstrated through comparison with data collected before the treatment (Kaluzna-Czaplinska and Blaszczyk, 2012). A case study showed an ASD boy with severe cognitive disability was treated with VSL#3 (a multi-strain mixture of 10 probiotics) for 4 weeks. The treatment relieved the GI symptoms and improved the autistic core symptoms (Grossi et al., 2016). In a recent clinical study, which is currently in progress, 100 preschoolers with ASD are being administered probiotics or a placebo for 6 months, and the results will provide new information regarding the clinical and neurophysiological effects of the probiotic treatment in children with ASD (Santocchi et al., 2016).

Probiotic treatments have a proven ability to normalize the microbiota and ameliorate gut symptoms; however, the available evidence for prebiotics is lacking. The term “prebiotics” refers to non-digestible oligosaccharides that induce the growth of beneficial bacteria (Rosenfeld, 2015). The prebiotic galactooligosaccharide (B-GOS) increases the levels of *Bifidobacterium spp*. in an *in vitro* gut model, as demonstrated through the analysis of fecal samples from children with ASD and controls (Grimaldi et al., 2017).

However, the positive roles of probiotics or prebiotics in humans are controversial. In summary, probiotic, and prebiotic treatment for ASD patients lack multicenter, large- sample, randomized controlled trial.

### Fecal microbiota transplantation (FMT)

Fecal Microbiota Transplantation (FMT) is an intervention in which the fecal microbiota from a healthy individual is delivered to a patient with a dysbiotic gut microbiota. FMT dates back to fourth-century China, when a traditional Chinese medicine doctor described an oral human fecal suspension that effectively cured food poisoning and severe diarrhea (Zhang et al., 2012). It is highly efficacious in the treatment of recurrent *Clostridium difficile* infections (CDI) (Vrieze et al., 2013; Lessa et al., 2015). FMT has been applied to the treatment of IBD and IBS, based on the speculation that FMT can normalize the gut microbiota in patients with IBD and IBS, and improve constipation symptom (100%) (Aroniadis and Brandt, 2013; Rossen et al., 2015). Thus, researchers are increasingly interested in using FMT to treat children with ASD. However, the safety of FMT should be considered. The potential adverse events of FMT include diarrhea, abdominal cramps, belching in the short term, mild abdominal discomfort/bloating, and transient low-grade fever (Kelly et al., 2015).

### Microbiota transfer therapy (MTT)

Microbiota transfer therapy (MTT) is a modified FMT protocol comprising 14 days of antibiotic treatment followed by bowel cleansing and the administration of a high initial dose of standardized human gut microbiota (SHGM) for 7–8 weeks. An open-label clinical trial showed that MTT improved both GI symptoms (e.g., constipation, diarrhea, indigestion and abdominal pain) and ASD-related symptoms and normalized the microbiota of ASD patients (Kang et al., 2017).

### Other potential therapies (e.g., diet and antibiotics)

Children with ASD tolerate a narrower range of foods and exhibit more feeding problems than children without ASD (Schreck et al., 2004). Specifically, children with ASD refuse more foods and exhibit a more limited food repertoire than typically developing children (Bandini et al., 2010). Many parents complain that their children with ASD are selective eaters. Children with ASD reject foods for various reasons, including problems with the presentation of the food, the use of certain utensils, and the inclusion of different types of food on the same plate (Schreck and Williams, 2006). Compared with controls, children with ASD ingest fewer fruits, vegetables, and proteins and have a significantly lower daily intake of potassium, copper, folate, and calcium (Sharp et al., 2013; Malhi et al., 2017). Food intake influences the composition of the gut microbiota (Wu et al., 2011). Decreases in carbohydrate intake decrease the levels of *Roseburia* spp. and *Eubacterium rectale* (Duncan et al., 2007). Furthermore, diet-induced alterations in the location and composition of the gut microbiota influence the serum metabolite levels (Tremaroli and Bäckhed, 2012). According to an open-label trial, participants with ASD who are treated with omega-3 fatty acids for 12 weeks exhibit significant improvements in social behaviors (Ooi et al., 2015). However, high-dose supplementation with omega-3 fatty acids do not affect children with ASD, as demonstrated by a recent study (Mankad et al., 2015). A gluten-free and/or casein-free (GF/CF) diet improves ASD behaviors, physiological symptoms, and social behaviors (Knivsberg et al., 2002; Millward et al., 2004; Pennesi and Klein, 2012). The ketogenic diet is a high-fat and low-carbohydrate diet and results in reductions in the total gut microbial counts, increased sociability, decreased repetitive behaviors, and improved social communication in an ASD animal model (Ruskin et al., 2013; Castro et al., 2016). Newell et al. found that the ketogenic diet reduces the total gut microbial abundance in cecal and fecal matter and normalizes the level of *Akkermansia muciniphila* in an ASD murine model (Newell et al., 2016). Children with regressive-onset autism who are treated with vancomycin, a broad-spectrum oral antibiotic, for a short period exhibit improvements in diarrhea and autistic behaviors (Sandler et al., 2000). Based on a prospective, double-blind, placebo-controlled trial, 3-month treatment with levocarnitine improves ASD symptoms in children with ASD (Geier et al., 2011). More clinical studies are needed to establish support for the use of dietary, antibiotic, and supplement treatments.

## Conclusion

In this review, we summarize the information from multiple studies showing that an abnormal gut microbiota is related to ASD. First, we reviewed the relationship between the gut microbiota and the CNS. Second, we defined the role of the gut microbiota in ASD. Finally, we described some potential therapies for modulating the gut microbiota in patients with ASD. Many recent clinical studies have shown that treatments that regulate the gut microbiota result in improvements in ASD symptoms (Critchfield et al., 2011; Tomova et al., 2015). However, well-designed research studies with more participants are needed to provide more evidence that supports the effectiveness of these treatments.

## Author contributions

All authors listed, have made substantial, direct and intellectual contribution to the work, and approved it for publication.

### Conflict of interest statement

The authors declare that the research was conducted in the absence of any commercial or financial relationships that could be construed as a potential conflict of interest.
